# The entanglement of individuation and explanation in the discovery of xenogastrulation

**DOI:** 10.1007/s40656-025-00706-1

**Published:** 2025-11-27

**Authors:** Andrew Bollhagen, Zachary J Mayne, Christa S Merzdorf

**Affiliations:** 1 Department of Philosophy, UC Davis, Davis, USA; 2 Department of History and Philosophy of Science, University of Pittsburgh, Pittsburgh, USA; 3 Department of Microbiology and Cell Biology, Montana State University, Bozeman, USA

**Keywords:** Individuation, Characterization, Explanation, Experimentation, Developmental Biology, Discovery

## Abstract

This paper analyzes an episode of scientific work that was prompted by observations of a novel defect in early embryonic development, which was unexpectedly induced in an experimental context and has tentatively been dubbed “*xenogastrulation*.” The researchers worked to *individuate* this as a novel phenomenon—both by distinguishing it from what it is not (e.g., exogastrulation) and by forming a positive conception of what it is—in order to facilitate further inquiry. Our analysis provides new insights into the role of explanatory reasoning in nascent experimental research programs. We argue that the researchers’ efforts to individuate the novel phenomenon were *entangled* with their efforts to explain it. By this, we mean that tentative answers to the individuative question, “*what is* it?” also served as tentative answers to the explanatory question, “*by what means* does it occur?” This case study therefore demonstrates that explanation need not wait until an explanandum has been clearly individuated but instead can be deeply entangled with the process of individuating the explanandum in the first place.

## Introduction

This paper analyzes an episode of scientific work that was prompted by observations of a novel defect in early embryonic development, which was unexpectedly induced in an experimental context and has been tentatively dubbed “*xenogastrulation.*” We offer an analysis of the pattern of observation and reasoning by which researchers *individuated* this as a novel type of developmental defect*,* both distinguishing it from what it is *not—*i.e. neither normal development nor *exogastrulation,* a classic defective phenotype—and attempting to formulate a positive conception of what it *is* in order to facilitate further empirical study.^1^

In developing our analysis, we draw on philosophical work that understands developmental biology to be organized in terms of *problems agendas* composed of *research questions* that specify values for key variables relevant to posing such questions in this field (i.e., degree of abstraction, temporality, spatial composition, variety, and connectivity) (Love, 2018). We also draw on philosophical work that identifies *marking and tracking* as a key practice in developmental biology (Griesemer, 2007, 2018). As we will show, in our case study, researchers exercise of this practice specified key spatial and temporal variables relevant to posing researching questions in the wake of their initial observations of the novel phenomenon.

Furthermore, we argue that the distinctive manner in which researchers engaged in marking and tracking to *individuate* this novel defective morphology involved making characteristically *explanatory* inferences relevant to determining the means by which the embryos they observed came to have the unique features that they marked. Concern with explanation persisted as researchers attempted to formulate a positive conception of the novel defective morphology that would not only answer the individuative question, “*what is* it?” but also provide a tentative answer to the explanatory question, “*by what means* does it occur?” Our main claim, therefore, is that, in the pattern of observation and reasoning that the researchers pursued, efforts to *individuate* a novel phenomenon were *entangled* with efforts to *explain* it.

We make two notes before offering a roadmap for the remainder of the paper. First, as our case illustrates, important moments in the kind of scientific work we analyze here can be quite fleeting, taking place only within the initial moments following observation and in subsequent lab meetings. To our advantage, two of the authors on this paper are researchers who participated directly in the discovery of xenogastrulation. The analysis we present would not have been possible if this were not the case. We suggest that the form of interdisciplinarity that this paper reflects is a valuable resource for increasing the resolution of philosophical analyses of active scientific research.

Second, this paper will also constitute the first published discussion of xenogastrulation as a developmental defect; however, we think it is crucial to emphasize that we are offering a philosophical analysis, not a scientific report, and so we do not intend to make any claims here about the biological significance of xenogastrulation. It could very well turn out to be an experimental quirk that leads to little more than the local episode analyzed here. But if, through continuing empirical investigation, xenogastrulation comes to have broader biological significance, this paper would represent a unique situation in which a philosophical journal hosts the initial discussion of a newly discovered biological phenomenon.

The remainder of the paper proceeds as follows. Section 2 discusses in turn each of the key terms in which we formulate our main thesis (“individuation,” “explanation,” and “entanglement”). Section 3 discusses in greater detail the philosophical resources on which we draw in developing our analysis of the case study (“marking and tracking,” and “problem agendas”). Section 4 presents relevant scientific background and the case study. Section 5 breaks the case down into two “key moments” and develops our argument by analyzing each key moment in terms of the framework we now introduce. Section 6 concludes.

## Individuation, explanation, and entanglement

This section details the senses in which we use the terms “individuation,” “explanation,” and “entanglement.” We will draw heavily on existing philosophical literature for each but will differentiate our own views where necessary.

### Individuation

First, consider two senses of the term “individuation” distinguished by Bueno et al. (2018).

- Practical individuation: The practical process in which scientists manipulate, target, track, present, or produce an individual by means of scientific procedures.
- Epistemic individuation: The cognitive process by which scientists identify, distinguish, and individuate a thing from its environment and other things (7).

Both are relevant to our analysis. Marking and tracking is a process of practical individuation that researchers in our case study deployed in order to identify and observe parts and processes that were characteristic of the defective embryos of interest. Our main focus, however, is on the broader task to which marking and tracking was put in our case study. This broader task is what Bueno et al. call “epistemic individuation.” In particular, we focus our analysis on the “cognitive process”—the pattern of observation and reasoning—by which scientists distinguished these “things” (the novel defective embryos they observed) from “other things” (normal and exogastrulation) and formulated a positive conception of *xenogastrulation* as an object of investigation in its own right.

It will now be useful to consider briefly, as a foil, another context in which philosophers have discussed the “cognitive process” of epistemic individuation, namely, in debates regarding sortal concepts.^2^ According to Freund and Grandy (2025), while there is no single “received view” on the matter, philosophers typically understand the roles of sortal concepts to include: defining the essence of thing (i.e., providing an answer to the question, “what is it?”); offering a principle of individuation that enables us to single out an entity; establishing criteria for identity and differentiation (i.e., specifying when something is the same as or different from a given object); and providing a persistence criterion, determining when something continues or ceases to exist.

One view of sortal concepts, sometimes called *psychosortalism* or *perception sortalism,* “holds that sortals are essential for perception, as they are thought to be the means by which objects are identified” (Campbell, 2002; Clark, 2006). On this view, for example, in order to perceptually individuate mugs, one needs to possess the sortal concept *mug* which provides the criteria required to distinguish it from its environment and other objects (Lowe, 2007 p. 514). Detractors from this view argue that the possession of sortal concepts is not a necessary condition on effective perception as there are other cognitive mechanisms that “direct conscious attention to objects based on spatiotemporal and property information. This form of individuation is shared by animals such as cats and dogs” (Freund & Grandy, 2025).

Our intention is not to develop a direct intervention into the literature on sortal concepts. We mention psychosortalism only briefly to note the ways in which it falls short in its capacity to analyze our case study. First, pyschosortalism understands sortal concepts as constitutive of the *means by which* cognizers achieve the *end* of epistemic individuation. In our case, however, it is clear that it was not by means of a sortal concept at all that the researchers individuated the novel defective embryos they observed. In fact, one might say that the means-end relation was the reverse. The pattern of observation and reasoning that we will analyze constitutes the means by which researchers achieved, in the end, a (tentative) positive answer to the question “what is it?” that philosophers might identify with a sortal concept, *xenogastrulation.*^3^

Second, we note that in the list of roles attributed to sortal concepts mentioned above, the function of answering the explanatory question, “by what means does it occur?” is not present. Yet, as we will see, pursuing answers to this question was an aim reflected in the researchers’ reasoning pattern as much as was the aim of answering the “what is it?” question and distinguishing it from other associated phenomena. Indeed, we argue that, in the pattern of observation and reasoning we reconstruct, these two aims cannot be separated analytically. As we put it later in our conclusion, it is positively ambiguous as to whether key moments in the pattern of observation and reasoning were aimed at individuating or explaining the novel phenotype. This ambiguity is expressive of our claim that individuation and explanation were “entangled” in this pattern of reasoning.

Finally—and this is another reason we do not intend our analysis as an intervention into the literature on sortals—we hesitate to refer to the positive conception of xenogastrulation that researchers in our case formulated as a sortal concept for the reason that its role in scientific practice exceeds those typically associated with sortal concepts. As we discuss later, the content of the “finished” concept (scare quotes indicating that the research is ongoing) inherits from the pattern of observation and reasoning involved in its development “entangled” individuative and explanatory content and, as can be observed in the list of functions philosophers generally attribute to sortal concepts, specifying the means by which, say, mugs come about is not among them.

The literature we intend to address more directly has been contributed to by a number of philosophers of biology who approach questions of individuation from a perspective that hews closely to the details of case studies of biological practice. Waters (2018) puts the contrast between the approach we follow and more traditional approaches to individuation as follows, “Biologists and philosophers often ask ‘what is an X?’ with an emphasis on the ‘is’... it is as if one can hear the stamping of feet upon utterance of the word “is” when we ask, what is a species?, what is an organism?, or what is a gene?” In contrast to asking for general, context-free criteria (the likes of which might be provided by a sortal concept) for what makes an X an X, Waters suggests that philosophers ask not e.g., “what *is* a gene?,” but “how do biologists individuate genes?” and “do their practices of individuation serve their purposes?” (p. 91).

Accordingly, in his own analysis, Waters considers how geneticists across the twentieth century have differentially individuated genes for different purposes of manipulation, prediction, explanation, and investigation. In the same practice-oriented spirit, McConwell (2024) discusses, among other things, how biologists individuate organisms by reference to “unique features” that enable field biologists to identify species “on the fly” and against the background of a noisy environment. Love (2018) offers an analysis of how developmental biologists individuate parts of embryos by “marking” them for the purposes of “tracking” them over the course of a developmental stage. Following suit, our analysis seeks to contribute to this literature a philosophical understanding of the pattern of observation and reasoning involved in individuating a novel developmental phenomenon immediately in the wake of its initial observation.

### Explanation

Turning now to *explanation*, there is a literature regarding the kinds of explanations that biologists formulate for developmental phenomena and philosophical debate over how to evaluate them (see, e.g., Weber, 2022).For example, some biologists and philosophers favor a *genetic* explanatory approach which works by “identifying changes in the expression of genes and interactions among their RNA and protein products that lead to changes in the properties of morphological features during ontogeny (e.g. size or shape)... “ (Love, 2022). Others prefer *physical* explanations according to which developmental phenomena occur by means of mechanical forces exerted on and by mesoscale materials as they undergo geometrical rearrangements (Forgacs & Newman, 2005).

As we will see, in making what we identify as explanatory inferences, the researchers in our case were reasoning in terms of mechanical forces exerted on and by mesoscale materials and also in terms of molecular and genetic mechanisms, which served as targets of intervention when they accidentally discovered the novel defect in question. We do not mean to claim that the specific terms in which their explanatory reasoning proceeded constitute *the* terms in which explanations in developmental biology always in fact, or even ought to, proceed. We mean only to claim that, in the pattern of observation and reasoning we analyze, appeals to the kinds of physical and molecular mechanical features that the authors in the paragraph above discuss functioned explanatorily in the researchers’ reasoning. We do assume that explanation in developmental biology is causal in a generic sense of that word (Parkkinen, 2014; McManus 2012) but otherwise remain neutral in debates over the proper categories in terms of which to formulate explanations in developmental biology. Indeed, according to Love (2008, 2018) the adjudication of these issues occurs *within* development biology and is part and parcel of what gives it, as a science organized according to problem agendas, its characteristic structure at a time. So, to maintain critical distance from these questions, we will say that to explain a developmental phenomenon is to give an account of the *means by which* it occurs, irrespective of internal disputes regarding the more determinate categories in terms of which to explicate those means.^4^

Thus, to make the contrast we need to draw, we will say that, while individuative questions take the form of “*what is an* X?,” explanatory questions take the form of “*by what means* does X occur?” The former is the kind of question which standardly requests an answer in terms of identity conditions—a set of features in virtue of which token Xs count as members of the *X* type.^5^ The latter poses a question to be answered in terms of, say, push–pull forces, biomolecular mechanisms, evolutionary forces, characteristic forms of human activity, etc.

### The entanglement of individuation and explanation

As a reviewer of this article helpfully points out, the practice of individuation is intimately related to broader scientific practices of description and characterization. While the relationship we explore in this paper—that between *individuation* and explanation—has not been directly analyzed, the relationship between *characterization* and explanation has been the subject of much philosophical commentary. It is useful, therefore, to consider our analysis in light of philosophically adjacent analyses of the relation between characterization and explanation (Colaço, 2018; Colaço, 2020; Bollhagen, 2021a, 2021b; Bechtel & Vagnino, 2022; Dresow & Love, 2025; Feest, 2017; Hauies 2023).^6^

While there are key differences between how philosophers understand the relationship between these two activities, they generally agree that characterizing a phenomenon is a matter of specifying an *explanandum*—a target of explanation—and that explaining a phenomenon specifies an *explanans.* The key point of contention relevant to our discussion pertains to the manner in which these two forms of scientific work are related. On what Dresow and Love (2025) identify as a would-be “received view” inherited from the New Mechanist philosophical tradition, characterization is merely a means to an ultimately explanatory end.^7^ Characterizations, on this view, tell scientists what they need to attend to and what exactly needs explaining. As Craver (2014) writes, “to characterize a phenomenon correctly and completely is a crucial step in mechanism discovery” (p. 128). Discussing the purpose of characterizing phenomena, Craver and Darden (2013) also give explanation the limelight, saying that characterizations “prune the space of possible mechanisms” (p. 62).

The “received view” allows that a phenomenon may be re-characterized as explanatory insights into the phenomenon are ascertained. Bechtel and Richardson (2022) write that phenomena are “reconstituted” because “our conception of what needs explaining... is shaped by the explanations and the models we develop” (p. 194). Craver and Darden agree, stating that “a purported phenomenon might be recharacterized or discarded entirely as one learns more about the underlying mechanisms” (p. 62). While mechanist philosophers recognize that characterizations can be rejected and modified in this way, *explaining* phenomena in terms of the mechanisms that produce them is, by and large, the focus of philosophical reflection. As Dresow and Love (2025) write, this way of thinking maintains that “... characterizations themselves are not objects of active scrutiny” and that “characterization is an activity separate from, and subordinate to, explanation” (p. 519).

In contrast, recent expositions have suggested that characterization work enjoys, at least to some degree, a life of its own. Bollhagen (2021a, 2021b; *in press*), for example, discusses a case in which researchers came to re-characterize a phenomenon within the context of an experimental program dedicated solely to characterizing, rather than explaining, the dynamics of the motor protein kinesin. Bechtel and Vagnino (2022) follow suit in their analysis of characterization work that led researchers to recognize membrane potentials and action potentials as distinct phenomena. Feest (2017) discusses experiments in psychology that, she argues, are better understood as aimed at further characterizing spatial memory rather than trying to explain it. Finally, based on their analysis of scientific efforts to characterize the “explosiveness” of the Cambrian Explosion, Dresow and Love (2025) conclude that “characterization is often done for its own sake and not solely for the purpose of facilitating explanation.” Indeed, this is the common theme marking recent philosophical departure from the “received view.” Scientists can characterize and re-characterize phenomena for different purposes depending upon their “theoretical and practical aims” (Colaço, 2020).

In the same language that we use to formulate our main claim, Dresow and Love (2025) describe the work of characterizing and explaining the Cambrian Explosion (CE) as “entangled.” To illustrate, they argue that characterizations of associated phenomena—e.g. the redox state of the Neoproterozoic ocean—that are potentially relevant to explaining the CE can be formulated by researchers in one discipline yet “grow the roster of explanatory resources” for researchers in other disciplines interested in explaining the CE. Their analysis, however, focuses on scientific work at a significantly greater spatiotemporal scale than our own analysis. As they write, “researchers engaged in characterization often belong to entirely different disciplinary communities than those engaged in formulating explanatory models” and “... scientists engaged in characterizing a phenomenon may rarely collaborate, or even communicate, with scientists interested in explaining that phenomenon.” The “entanglement thesis” that they endorse emphasizes the interdisciplinarity involved in the complex ways in which characterization and explanation work interact and mutually influence each other across the spatiotemporally vast distribution of labor characteristic of research on the CE.

Our analysis, in contrast, focuses on scientific work on a much shorter timescale, involving mainly the researchers working together in a single developmental biology lab. Further, our use of the term “entanglement” is different from Dresow & Love’s in the sense that, for them, “entanglement” refers to the interaction between two analytically distinguishable forms of scientific activity—characterization and explanation. For us, on the other hand, “entanglement” refers to the fact that it is a matter of positive ambiguity whether the pattern of observation and reasoning that we analyze is individuative or explanatory in nature.^8^

In sum, recent philosophical analyses attribute a degree of methodological autonomy to characterization vis-à-vis explanation denied to it by the “received view.” All of these recent views share with the “received view,” however, the idea that these two forms of scientific work can be analytically distinguished. We acknowledge that this is very often the case, and we do not mean to offer any general challenge to the claim that important aspects of scientific practice lend themselves to analysis in terms of such a distinction. We mention this literature not to offer a challenge but to throw into relief the distinctively “entangled” character of the scientific work we analyze in our case study.

This literature is of further use because it enables us to note the possibility that some of the work we identify here as “individuation” may also count as “characterization” work according to philosophical construal of it in the literature mentioned above (especially the work that was done later in our case study, once the novel phenomenon had already begun to be individuated and had been granted a name). Therefore, insofar as philosophical analyses of characterization can be transposed into our analysis of individuation, it would follow that the activities of characterization and explanation are entangled to the point of analytic inseparability in our case study. To that extent, then, this article could be read as a contribution to the literature on characterization and explanation as well as a contribution to the philosophical literature on practices of individuation in the life sciences.

## Marking, tracking, and problem agendas

In developing the analysis of our case study, we draw upon philosophical work that (1) characterizes developmental biology as organized in terms of “problem agendas” and (2) identifies “marking” and “tracking” as key epistemic practices in developmental biology (Griesemer, 2007; Love, 2018). In this section, we introduce the basic elements of our analysis and sketch the argument that we develop over the following two sections.

First, according to Love (2008, 2014, 2018), rather than being structured by an overarching theory of development, developmental biology is organized in terms of “problem agendas” built around “generally delineated phenomena”—i.e. differentiation, specification or pattern formation, morphogenesis, and growth. On Love’s view, each general phenomenon can be further subdivided into specific research questions that embody the values of at least five different variables: degree of abstraction, temporality, spatial composition, variety, and connectivity. As Love (2018) illustrates:

research questions oriented around events in zebrafish gastrulation are structured in a way that differs from research questions oriented around vertebrate neural crest cell migration because they involve different values for the five variables: degree of abstraction (zebrafish vs. Vertebrates), temporality (earlier vs. later), spatial composition (tissue layer interactions vs. a distinctive population of cells), variety (epiboly vs. epithelium to mesenchyme transition), and connectivity (gut formation and endoderm vs. organogenesis and ectoderm/ mesoderm). (pp. 171–172)

According to this view, these tools for formulating local research questions within a broader problem agenda are sufficient for researchers in the field to pursue productive empirical inquiry in the absence of an overarching theory of development.

We do not take issue with this view. In fact, we will later draw on it to illustrate how considerations of temporality and spatial composition figured into researchers’ efforts to individuate the novel phenomenon they observed. We reiterate, however, that in the quotation above, the phenomena about which research questions are formulated within a problem agenda are presented as *already* individuated. In other words, the question of “what is zebrafish gastrulation?” or “what is neural crest cell migration?” are presented as having been answered at least to a degree sufficient for them to constitute targets about which further research questions can be formulated in terms that give values to the five variables that Love mentions. We point this out to throw into relief the fact that our case represents a phase of inquiry further upstream than what is explicitly represented in the quotation—so far upstream, in fact, that our episode begins prior to researchers even giving their novel phenomenon a name like those used in the quoted passage (e.g. “gastrulation,” or “neural crest migration”).

Second, the phrase “marking and tracking” refers to a variety of techniques that biologists use to follow processes of interest. As Griesemer (2018) explains, scientists track “a process by observing (mental marking or taking note) or experimenting (“physical” marking by perturbation or alteration) and thereby create ‘traces’: physical remnants that can be used as representations of the state of a marked process at multiple times. A sequence of these traces represents the process as it was tracked.” The basic idea of tracking is simple. Indeed, according to Griesemer (2007), “[tracking techniques] are only more systematic and disciplined versions of what casual observers do on a lazy summer afternoon when they track the flow of a river by watching the movement of a leaf floating downstream” (p. 379). Such a casual observer would “mentally” mark the leaf and track the flow of the river, perhaps discerning something about the pattern of the currents or the rate at which it flows. In biology, “physical” marking by, say, putting fluorescent labels (markers) on proteins so as to track their movement through a cell, or staining a particular tissue within an embryo so as to track its dynamics during a developmental stage, are clearly more technically sophisticated than leaf-watching. But the purpose is the same—to track a process that would be difficult to follow otherwise. To give another intuitive example, the tracking label on a FedEx package is a marker and the notifications a sender receives as the package arrives at various waypoints are “traces” which together represent the “tracked” delivery process.

Again, the aim of this paper is to offer an analysis of the pattern of observation and reasoning by which researchers in our case study individuated a novel developmental defect that they came to call *xenogastrulation*, both distinguishing it from what it is *not*—normal development nor exogastrulation—and formulating a positive conception of it in order to facilitate further empirical research. As our analysis of the case study will show, researchers supported this pattern of observation and reasoning by “marking” unique features of the phenomenon in order to “track” developmental processes and organize a conception of the novel phenomenon that specified values for the variables of *spatial organization* and *temporality* that Love discusses. Thus, our case study provides support for the usefulness of these ideas in analyzing practice in developmental biology.

We claim, however, that the manner in which researchers in our case marked and tracked is distinctive in a way that is significant for the development of our overarching argument. In our case, researchers “marked” unique features of the novel phenomenon and then treated *those very marks* as “traces” representing a process that, researchers reasoned, must have occurred given the presence of the marked feature. It might sound odd to claim that, in our case, marks and traces coincide in this way. After all, as the FedEx example illustrates, a mark is one thing (e.g., a tracking label on a package) and a trace another (e.g., a notification in your email inbox). It is distinctive of our case study, however, that researchers indeed marked unique features of the novel phenomenon and used *those very features* as “traces.” Indeed, it is precisely the fact that, in this pattern of observation and reasoning, the relevant features are ambiguously both marks and traces that renders the pattern one in which efforts to individuate the novel phenomenon were entangled with efforts to explain it.

This is not the only way, however, in which explanatory concerns pervaded their efforts to individuate the novel defect. After distinguishing it from what it is *not* and dubbing it “xenogastrulation,” researchers worked to formulate a positive conception of the novel defect that would facilitate its further empirical study. What they achieved was an answer to the individuative question, “what is xenogastrulation?” that doubles as a tentative explanation—that is, an account of the *means by which* xenogastrulation occurs. This is unsurprising in light of the fact that, as we intend to show, the pattern of observation and reasoning that these researchers pursued in *individuating* the novel phenomenon was distinctively explanatory as well.

We make good on these claims in Sect. 5**.** There, we break the case study down into two key moments: (1) what it is *not* and (2) what it *is.* Each key moment illustrates our primary claim, namely, that individuation and explanation were entangled in the pattern of observation and reasoning that the researchers pursued in the immediate wake of their initial observations of the phenomenon. Before that, however, we present our case study as well as some relevant scientific background in Sect. 4.

## Scientific background and the discovery of xenogastrulation

Our case study begins amidst a research program at the Merzdorf laboratory, which is aimed at understanding the molecular mechanisms driving *convergent extension*. Convergent extension is a crucial developmental process that helps establish the foundations of the nervous system in early embryos by forming a structure known as the *notochord*.

**Fig. 1:**
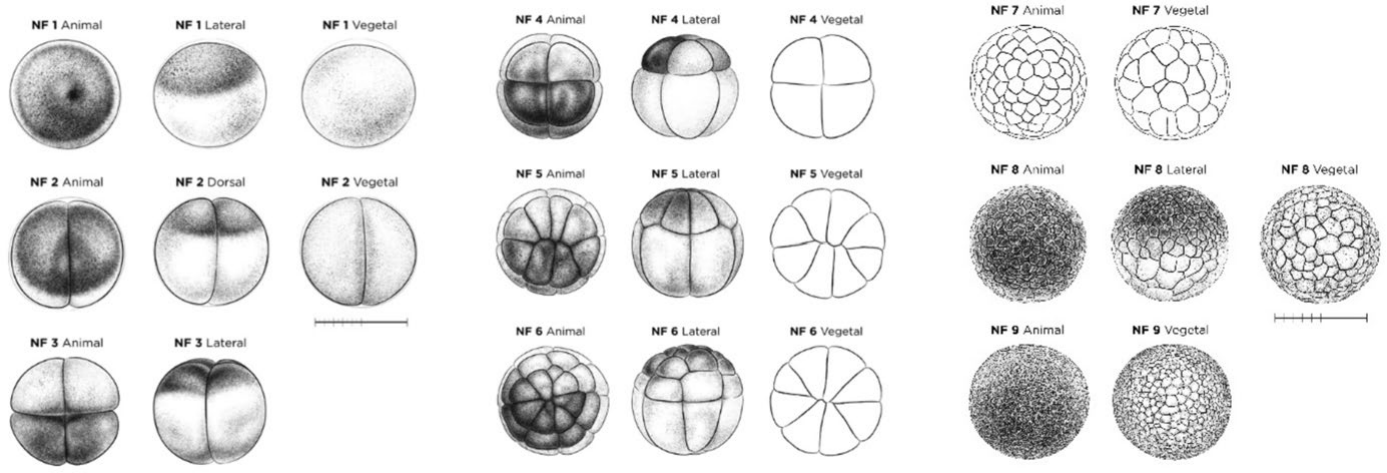
*X. laevis* embryo progressing through the first nine Nieuwkoop-Faber (NF) stages (NF 1–9). Drawings show the embryo as seen looking down through a microscope at the upward-facing *animal pole*, turned on its *lateral* or *dorsal* side, and turned over to show its downward-facing *vegetal pole*. Re-printed from Xenopus illustrations © Natalya Zahn (2022) (www.xenbase.org RRID:SCR_003280)

The investigators use *Xenopus laevis* as a model organism. *X. laevis* is a classic model organism in developmental biology, in part because the oocytes are quite large (~ 1 mm in diameter), making them easy to experimentally manipulate and observe (Fig. 1).

### Background: gastrulation, convergent extension, and neural induction

**Fig. 2:**
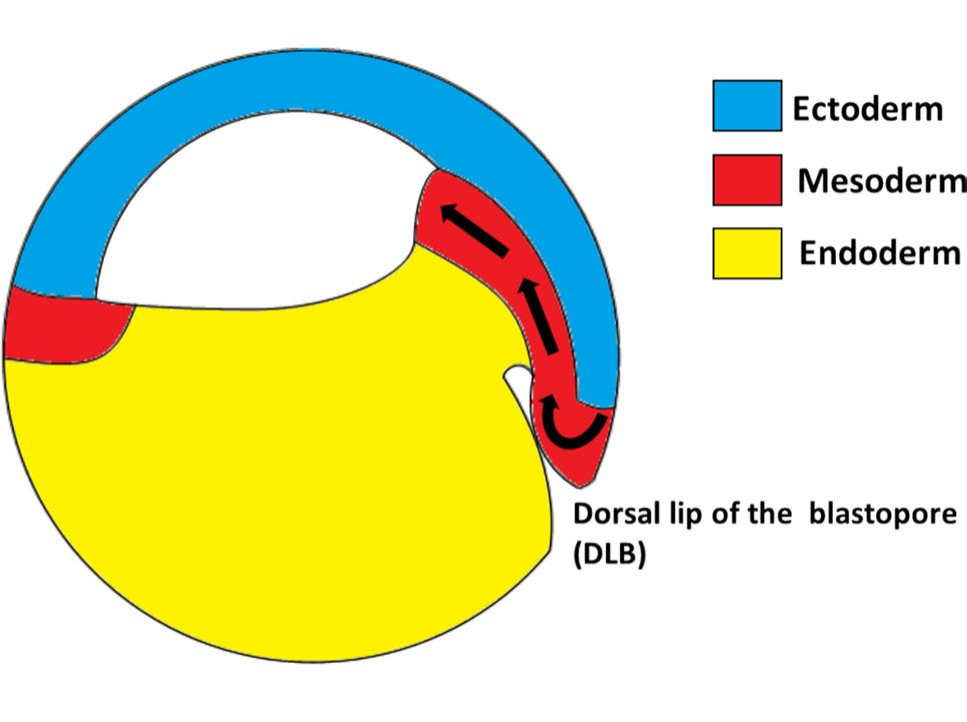
Lateral cross-sectional view of the involution of dorsal mesoderm through the dorsal lip of the blastopore (DLB) during gastrulation in *X. laevis*

At approximately 9 hours post-fertilization (hpf), *X*. *laevis* embryos reach the end of the *blastula* stages (stages 7–9) and begin the process of *gastrulation* (stages 10–13). The initiating event of gastrulation is when a population of cells from the presumptive mesoderm invaginates and involutes from the surface of the embryo into its interior, forming the *dorsal lip of the blastopore* (DLB) (Fig. 2). This population of cells then migrates along the roof of the embryo’s interior (Huang & Winklbauer, 2018).

**Fig. 3:**
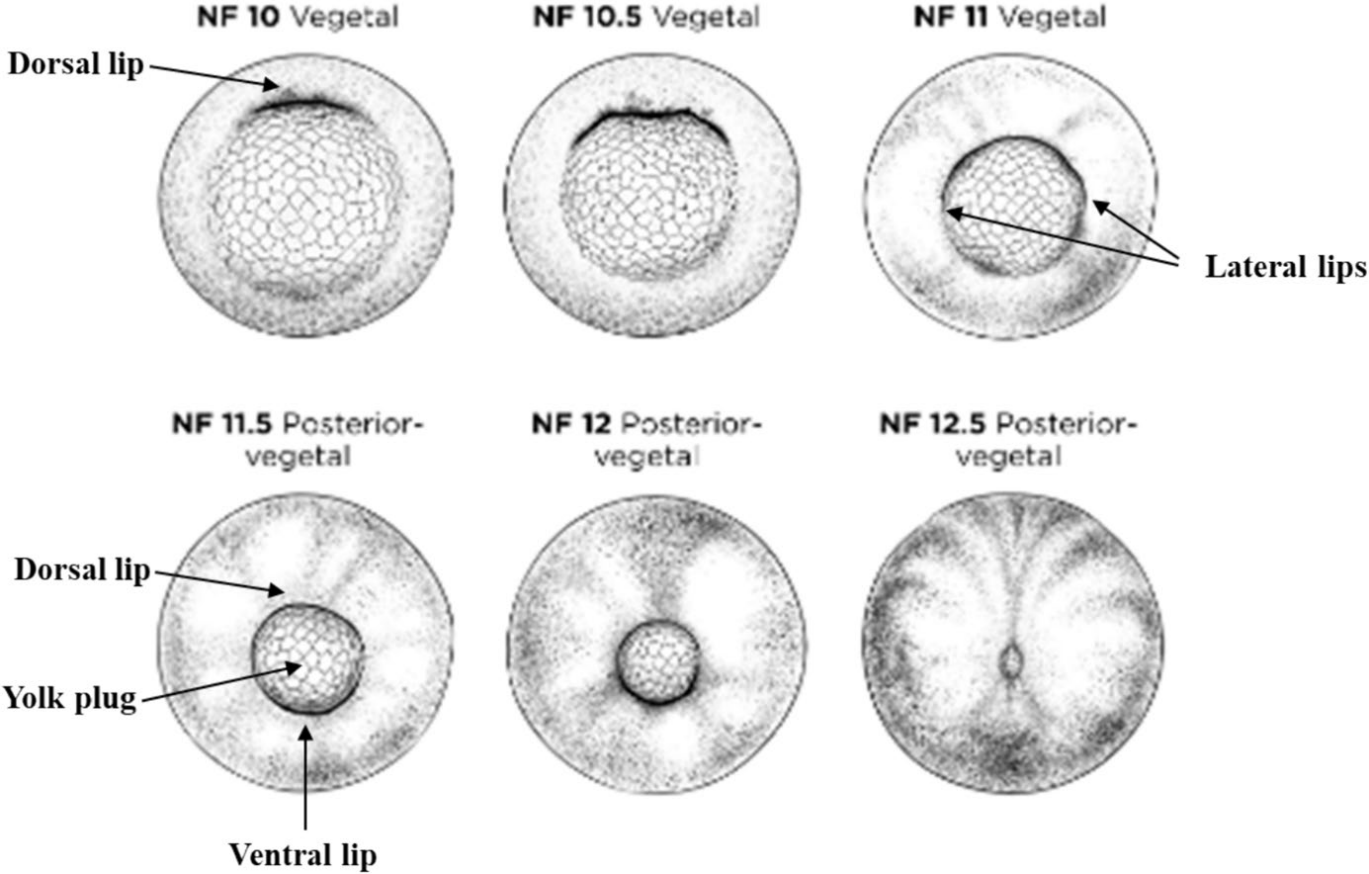
Vegetal view of the formation and constriction of the blastopore (indicated with a darker line) through gastrulation (stages 10–12.5 shown). Altered version of Xenopus illustrations © Natalya Zahn (2022) (www.xenbase.org RRID:SCR_003280)

These initial involuting cells become specified as *dorsal mesoderm*. As gastrulation proceeds, more cells involute at the edges of the dorsal lip, then at the lateral and ventral edges, so that the blastopore forms a growing crescent and then ultimately a closed ring. The blastopore ring then constricts as involution proceeds all the way around its circumference (Fig. 3).

**Fig. 4:**
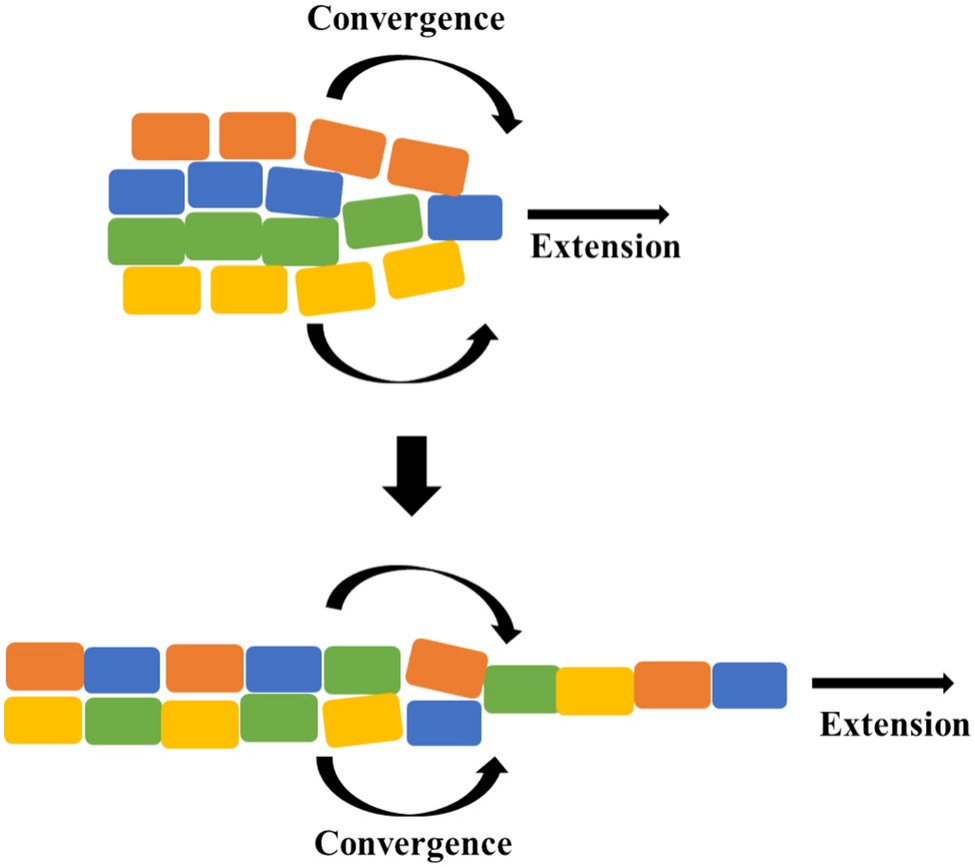
Schematic representation of convergent extension

As they migrate interior to the embryo after involution, the dorsal mesoderm cells undergo convergent extension, which can be analogized to a diffuse crowd of people organizing into a long, thin queue, or the merging of several lanes of traffic (Fig. 4). This process forms the notochord, a solid rod that extends along the anteroposterior (head-to-tail) axis of the embryo.

The notochord releases signals vertically that are received by the overlying ectoderm, inducing this region of ectoderm to become *neuroectoderm*. This process is known as *neural induction*. The neuroectoderm forms the neural plate, which then folds into the neural tube, from which the central nervous system is derived (Gilbert & Barresi, 2018, pp. 415–426). It will be worthwhile now to present some historical background on the discovery of vertical signaling in neural induction, as it involved the experimental use of a classic form of defective embryonic development called *exogastrulation*, which makes an important appearance in our case study.

In the 1920s, while working in the lab of Hans Spemann, Hilde Mangold ran a series of now-famous experiments in which she transplanted tissue from the dorsal lip of the blastopore from the embryo of one amphibian and grafted it into the ventral marginal zone of another at the same developmental stage as the donor (Spemann & Mangold, 1924). The result was the formation of a secondary body axis that was almost identical to the primary body axis. The presumptive mesoderm cells from the dorsal lip of the blastopore came to be known as the “Spemann-Mangold organizer,” indicating its crucial role in ‘organizing’ embryonic development.

Johannes Holtfreter, who was a graduate student in Spemann’s lab when Mangold discovered the organizer, found in 1933 that he could induce a defective developmental phenotype if he removed the membranes that surround early gastrulae, also known as vitelline envelopes, and placed them upside-down in a hypertonic saline solution (Holtfreter, 1933). Under these conditions, instead of involuting into the embryo, the organizer migrates in the opposite direction, pulling away from the rest of the embryo. The result is a dumb-bell shaped *exogastrula*.^9^

More important than the dumb-bell shape is the fact that, in exogastrulae, the organizer is located out on the protruding tissue. It remains connected to ectoderm but in a planar spatial relation rather than in the vertical relation characteristic of normal gastrulation. Holtfreter’s observation that there is no neural induction of ectoderm in exogastrulae led him to reason that signals from the organizer must be communicated from mesoderm to ectoderm *vertically*, and that *planar* signaling between the tissues is insufficient to induce neural tube formation.

Since Holtfreter’s experiments, new questions have been raised about the possibility of neural induction via planar signals in exogastrulae (Altaba et al. 1992), but it is difficult to rule out the possibility that, in cases of putative planar signaling, transient involution occurs to enable vertical signaling. Only insofar as exogastrulation is *total*—i.e. presumptive mesoderm entirely fails to involute—can researchers be confident that whatever neural induction has occurred must be due to planar signaling from the organizer region. These concerns are not idle, as it is widely known that some neural induction occurs in *partial* exogastrulation, although it usually is incomplete and leads to neural tube defects (NTDs) of varying severity.

So much for relevant background. We turn now to our case study.

### A novel gastrulation defect

In researching the mechanisms of convergent extension, scientists in the Merzdorf lab sometimes use in situ* hybridization*, a prominent technique in developmental biology, to “mark” the notochord and “track” its behavior as it undergoes convergent extension (Love, 2018). More specifically, the researchers bleach the embryos and stain cells expressing *chordin*, a gene expressed uniquely in dorsal mesoderm, with purple dye (a marker). They perform this technique on embryos that have been fixed at various time points in gastrulation and image them to creating a series of “traces” by which to track the process of convergent extension.

In these experiments, researchers make molecular manipulations to perturb biological pathways, track the resultant behavior of the marked dorsal mesoderm during convergent extension, and compare the marked tissue across control and experimental embryos. In drawing these comparisons, the researchers deploy a statistical notion of normal development.^10^ They establish the statistics of notochord size, especially length and width, in the control embryos to represent normal notochord formation. This serves as a standard of comparison with which they formulate characterizations of notochord in experimental embryos in terms of their departure from normality. On the basis of these comparisons, the researchers draw inferences regarding the molecular mechanisms driving convergent extension. For example, if the intervention inhibits a molecule known to participate in the Wnt-signaling pathway and this leads to the formation of shorter and wider notochords, this might suggest that that molecule plays an active role in the cellular processes of convergent extension.

**Fig. 5:**
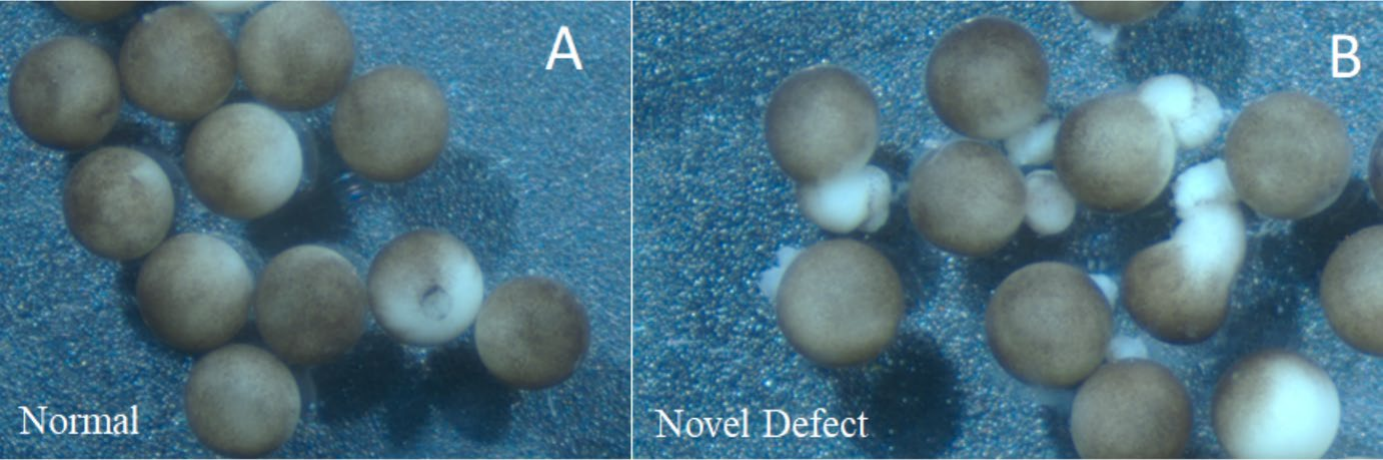
Normal gastrulating *X. laevis* embryos at stage 12 (**A**) next to the aberrant embryos of equivalent staging (**B**)

It was in the course of doing such research that an unusual phenotype was observed among several embryos from a particular experimental condition (Fig. 5).^11^ Researchers made molecular interventions in non-canonical Wnt-signaling pathways and measured their effects on convergent extension. Ordinarily, manipulations of non-canonical Wnt signaling pathways do not affect the (spherical) shape of the embryo, although they do affect the shape of the notochord. In this case, however, a number of embryos in the experimental group were observed to have long protrusions, giving them a dumb-bell shape (Fig. 5B).

**Fig. 6:**
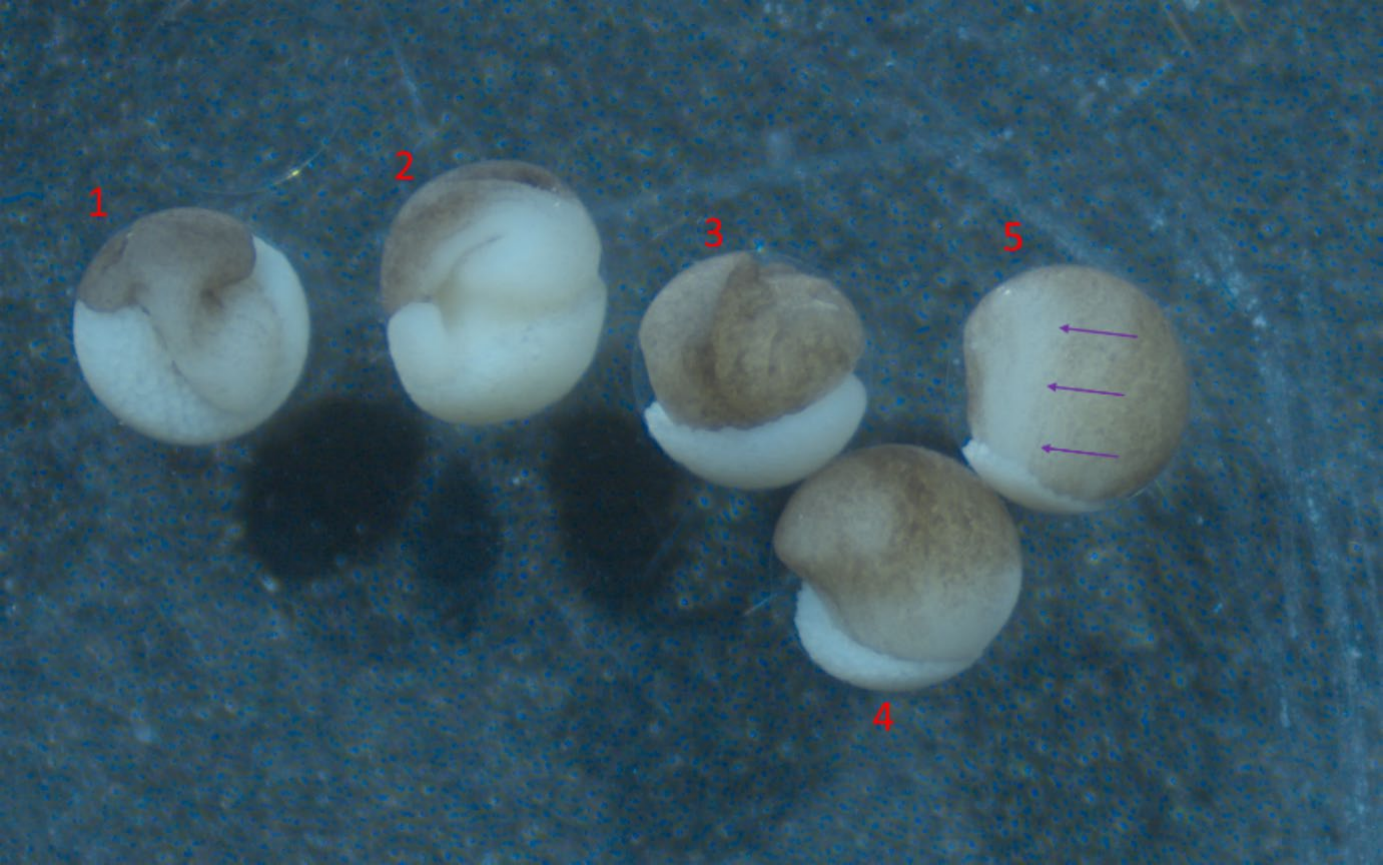
Exogastrulae of varying severity. 1–3 are total exogastrulae with no neural induction. 4–5 are partial exogastrulae; 5 shows clear formation of the neural plate, indicated with purple arrows. Note that the distinctive “dumb-bell” shape of these particular exogastrulae is suppressed by the presence of the vitelline envelope, a glycoprotein membrane which serves to hold the embryo in a spherical shape

**Fig. 7:**
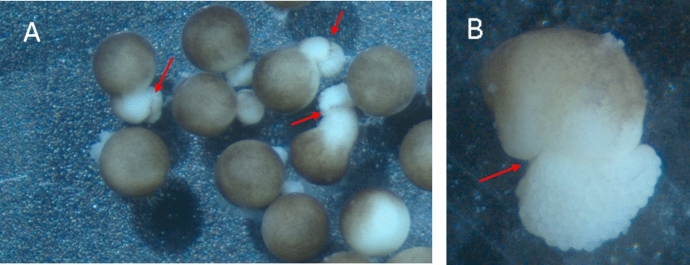
Blastopore positions indicated with red arrows. **A** Novel defect; **B** partial exogastrulation

The defective embryos were unlike any the researchers had seen previously, and they lacked a suitable category by which to identify them. Based on their dumb-bell shape, one researcher initially suggested that the phenomenon looked like “*a strange form of exogastrulation*” (Fig. 6). This proposal was swiftly rejected, however, in light of another researcher’s prompt response: “no, look at the position of the blastopore.” In fact, rejecting “exogastrulation” upon noting the position of the blastopore was not an occurrence unique to the moments immediately following the researchers’ observations. Interestingly, this same pattern recurred in conversation several times independently as the researchers discussed these findings at conferences and via e-mail correspondence with developmental biologists from other organizations. The general pattern can be summed up as follows: Upon initially hearing of the phenomenon and before studying images like Fig. 7A closely, the developmental biologist naturally thinks that the phenotype might be a kind of exogastrulation; then, upon noting the position of the blastopore, they decisively reject that description. Why?

We offer a fuller answer to this question in the next section. For now, suffice it to say that developmental biologists understand that the blastopore is the site of the dynamic process of *involution—*the process by which mesoderm adopts its location under ectoderm in normal gastrulation. The “exo” in “exogastrulation” refers to the fact that this process does not occur and so mesoderm remains *outside*, migrating away from the rest of the embryo. In the embryos showing the novel defect, the blastopore migrates down the elongation such that the majority of the elongation lies *between* the blastopore and the rest of the embryo. This indicates to researchers that involution has *already* occurred and, therefore, that the elongation is produced *after* involution occurs, rather than involution not occurring at all as in exogastrulation. It further indicates that the elongation is contained *within* the normal boundaries of the embryo, not extruding *outside* of it as it does in exogastrulation, and that mesoderm is located *under* ectoderm as in normal gastrulation.

Thus, for developmental biologists, the position of the blastopore on the novel defect is replete with spatio-temporal information. Indeed, noting the position of the blastopore led researchers to organize a conception of this novel phenomenon that assigns values, indicated by the italicized terms above, to the variables of temporality and spatial composition that Love (2018) takes to be central in formulating research questions in developmental biology. So, as we discuss further in our conclusion, it was not merely on the basis of the position of the blastopore per se that researchers rejected “exogastrulation.” Rather, noting the position of the blastopore catalyzed the deployment of researchers’ background knowledge by which they organized a rich conception of the phenomenon that assigned values to a constellation of spatial and temporal variables which depart from the values characteristic of exogastrulation. We emphasize, however, that this is not the whole story and that we return to this moment in the next section.

**Fig. 8:**
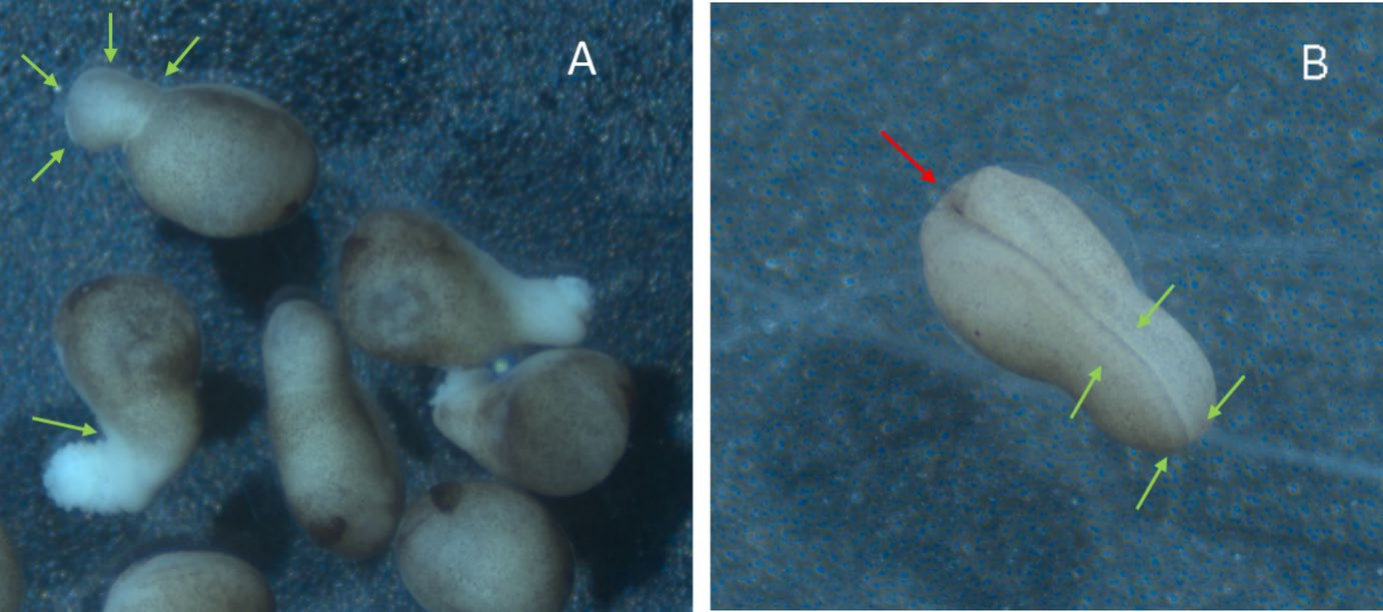
Successful neural tube formation along the elongation of embryos with the novel developmental defect (indicated with green arrows): **A** stage 17–19 embryos; **B** stage 18 embryo showing a neural tube defect (NTD), indicated with red arrow

Another key finding that cemented the rejection of “exogastrulation” was the observation of robust neural induction along the elongation (Fig. 8). Since, as we discussed above, vertical signaling from dorsal mesoderm is responsible for neural induction of overlying ectoderm, the observable presence of neural induction in ectoderm offers an indication that dorsal mesoderm has successfully involuted to its proper location beneath the ectoderm, albeit in an embryo exhibiting an unusual elongation.

Shortly after the researchers’ initial observations and subsequent rejection of “exogastrulation” (even a strange form of it), one researcher tentatively offered a new name for it: “*xenogastrulation*.”^12^ At the moment of its initial proposal, this name served as a useful placeholder for conceptually distinguishing this novel developmental defect from both normal gastrulation and exogastrulation in lieu of a positive conception of this novel defect in its own right.

That said, the researchers’ efforts at individuating this novel phenomenon did not stop at merely determining that what they now called “xenogastrulation” was *not* normal gastrulation nor exogastrulation. Rather, they sought to provide a positive answer to the question, “what is xenogastrulation?” Researchers put forward a number of positive formulations. The first of these was that xenogastrulation is “convergent extension of the entire embryo.” The researchers knew that the non-canonical wnt-pathways they perturbed in the embryos that xenogastrulated are causally connected with convergent extension. Indeed, that is precisely why they were perturbing them in the first place. Thus, when the embryos bulged out into their characteristically long and thin morphology, it was natural to diagnose it in terms of some intervention-induced alteration of convergent extension. Additionally, the appeal of “convergent extension of the entire embryo” was due to the fact that the shape of the whole embryo was lengthening and constricting, as does the dorsal mesoderm while undergoing convergent extension to form the notochord.

Through further discussion that we revisit in the next section, researchers in the Merzdorf lab drifted from “convergent extension of the whole embryo” to a formulation that included a better sense of how to move forward with empirically investigating this phenomenon: “a mechanical distortion of the whole embryo due to hyperactive convergent extension of dorsal mesoderm.” Whereas it was unclear what could be done empirically to test “convergent extension of the entire embryo,” the researchers knew what to look for to vet the adequacy of “a mechanical distortion of the whole embryo due to hyperactive convergent extension of dorsal mesoderm.” In particular, they knew that they could look at the shape and location of the dorsal mesoderm—the tissue that undergoes convergent extension—to see whether it was excessively long and thin, extending down into the elongation.

The researchers decided to look through their old in situ* hybridization* (ISH) data to see if they had previously recorded any images of the dorsal mesoderm in xenogastrulae without noticing it.^13^ As we mentioned above, in their ISH experiments, the researchers bleach the embryos and “mark” notochord by staining cells expressing the *chordin* gene with purple dye. Since *chordin* is uniquely expressed in the dorsal mesoderm, this allows visualization of the notochord.

**Fig. 9:**
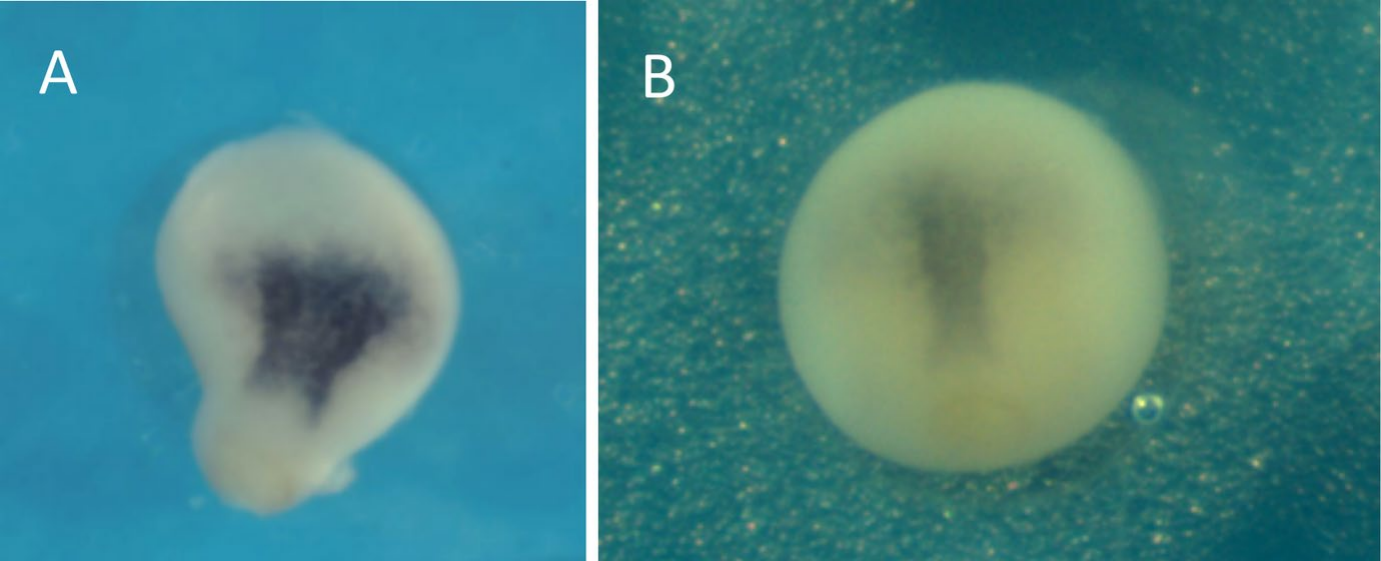
Stage 12 embryo showing xenogastrulation elongation (**A**) compared to a normal stage 12 embryo (**B**). Purple color from in situ hybridization indicates *chordin* expression, which is a gene uniquely expressed in the dorsal mesoderm

The researchers found at least one embryo in the old ISH data with moderate elongations of the sort seen in xenogastrulation. These data turned out to be somewhat less clear than the researchers had hoped, but they were nonetheless indicative that “a mechanical distortion of the whole embryo due to hyperactive convergent extension” was not apt. The dorsal mesoderm was not noticeably over-extended in the moderate xenogastrula compared to a normal embryo of the same stage, and the xenogastrula’s dorsal mesoderm was somewhat wider than normal (Fig. 9).^14^ If anything, the latter would typically indicate a reduction in convergent extension, as had been observed in experiments in which convergent extension was intentionally knocked down. Furthermore, the dorsal mesoderm was not obviously extending down into the elongation, as might be expected if convergent extension was driving the elongation outward.

Although the researchers in the lab did not entirely rule out “a mechanical distortion of the embryo due to hyperactive convergent extension in dorsal mesoderm” in light of these observations, they attempted to formulate alternative answers to the question “what is xenogastrulation?” but with little consensus. Recalling past experiments in which they had seen dorsal mesoderm extend without converging, the researchers then considered that, in xenogastrulation, dorsal mesoderm might be extending without converging, which could perhaps result in an unusual distribution of mechanical forces that could push the protuberance outward. This alternative description might be formulated as “a mechanical distortion of the whole embryo due to normal or slightly hyperactive *extension* of dorsal mesoderm without any *convergence*.”

With these alternatives on the table, researchers in the Merzdorf lab are currently planning to induce more embryos to xenogastrulate in order to collect more ISH data at different time-points in hopes of characterizing in more precise detail how the dorsal mesoderm is changing its shape and localization during xenogastrulation. The researchers agree that, if the data in Fig. 9 turn out to be robust and reproducible, “a mechanical distortion of the embryo due to hyperactive convergent extension” would have to be rejected. At present, this is the most direct “hypothesis-testing” experimentation that the lab is planning. The rest is exploratory. They plan to use markers for genes other than *chordin* to determine the identity of the cells in the elongation.

Also, in light of prior observations to the effect that the elongations of xenograstulae burst through the vitelline envelope prematurely, the researchers plan to remove the vitelline envelope in order to observe xenogastrulation with and without its presence. As the researchers have put it, this will help answer the question of “what does the xenogastrulating embryo ‘want’ to do without the vitelline envelope restraining it?” Finally, researchers plan to mark various tissues to track the development of their spatial and temporal organization in xenogastrulation. This could help identify other tissues that may be either distorted or perhaps even driving the distortion of the embryo. Although researchers believe the likelihood is low, experiments of this sort might reveal that, in xenogastrulation, the spatial relationship between tissues might turn out to be somehow different from how they are in normal gastrulation. Once researchers succeed in positively individuating xenogastrulation, mechanistic explanations of the phenomenon can be sought by perturbing key molecular pathways, most notably non-canonical Wnt pathways.

## Key moments

As we have stated, our goal is to offer a philosophical analysis of the pattern of observation and reasoning that researchers pursued immediately following their initial observations of the novel defect. In this section, we break down the case study into two key moments and analyze each in turn.

1. What it is *not:* This key moment includes the initial observations of the novel phenomenon, the rejection of “exogastrulation,” and the proffering of the name “xenogastrulation.”
2. What it *is:* “Convergent extension of the whole embryo” and its rejection in favor of “a mechanical distortion of the whole embryo due to hyperactive convergent extension of dorsal mesoderm,” leading to consulting old ISH data.

### What it is *not*

As we discussed above, the researchers’ rejection of “exogastrulation” followed a pattern common to both the immediate discussion around the microscope as researchers made their initial observations of the novel defect, and as the researchers discussed their findings with others via email and at conferences. Upon first seeing the novel defect, developmental biologists are inclined to identify it as exogastrulation (or a strange form of it). Then, after noting the position of the blastopore, they immediately reject that proposal.

We presented an aspect of our philosophical analysis of this pattern above. In rejecting “exogastrulation,” researchers did not do so merely on the basis of the position of the blastopore per se*.* Rather, noting the position of the blastopore catalyzed the deployment of researchers’ background knowledge which organized a conception of the phenomenon that assigned values to a constellation of variables pertaining to temporality and spatial composition (Love, 2008, 2018). Here, we bring to light another layer of observational and inferential complexity that is involved in this key moment by analyzing researchers’ rejection of “exogastrulation” in terms of the practice of *marking* and *tracking*. (Griesemer, 2007).

In order to provide contrast, however, it will be useful to first go into some detail regarding McConwell’s (2024) analysis of individuation practices in which scientists treat unique features of organisms as *cues* for species identification. The contrast between how scientists treat features of organisms as “cues” on McConwell’s account and how, on our account, researchers treated the unique feature (position of blastopore) of the novel defect as both a “mark” and a “trace” helps to elucidate the way in which individuation and explanation were entangled in the pattern of observation and reasoning we aim to analyze in this section.

McConwell’s analysis “addresses how biologists use unique features as cues to distinguish individual organisms as instances of their type while conducting fieldwork” (p. 12). To serve as a “cue,” a feature need not be of the sort that would figure in a set of criteria in terms of which a more formal taxonomy might be formulated. For instance, “smelling like rotten fruit upon destruction” is a unique feature of *Tapinoma sessile* (a species of house ant). This “cue” is indeed useful for identifying individual members of the species in the field but is not the kind of feature around which biologists would construct a formal taxonomy.

According to McConwell, decisions about which features to treat as cues (e.g. to cite in field guides) are made not on the basis of concern for systematic taxonomy but for their usefulness in facilitating species identification “on the fly” and “against complex environmental contexts” (p. 29). Individuating *Tapinoma sessile* from other similar looking ant species in the wild is an example. Further, she discusses how cues, as features of organisms that “stand out best and are easiest to identify” function like “mneumonics that can help a student... retain information for tests,” and that they “are passed down to students as prominent, distinguishing characteristics of organisms” (p. 14).

It is easy to imagine how cues would function in pedagogical contexts. To develop the example we alluded to above, if a number of ant species are present in an environment, an instructor might tell a student the “cue” that *Tapinoma sessile* “smells like rancid fruit upon destruction.” The student might then destroy select individuals and identify those that exhibit the cue as belonging to that species. Or, having studied a set of cues, students may be presented in the classroom with images of organisms and are told to label them according to their species. In this context, students would compare the images, searching for the relevant cues, and label accordingly. In general terms, according to McConwell, cues are used to facilitate species identification by means of a comparative pattern of observation and reasoning that involves judgements of the form: “x lacks characteristic y or perhaps has some feature that other non-x’s don’t have” (p. 12).

Analyzing the pattern of observation and reasoning that researchers in our case pursued in these terms is a non-starter. In our case, there was no antecedently specified “type” such that the unique features of the novel phenomenon could serve as cues for identifying the novel defective embryos as “tokens” thereof. That said, consider what it would look like if one tried. First, the researcher who makes the initial observation asks, “what is this? A strange form of exogastrulation?” A second researcher responds, “no, look at the position of the blastopore.” The proposed analysis would take the second researcher to be treating the blastopore as a cue, pointing out that the novel defect has some feature that exogastrulation does not have. Thus, on this analysis, the mere presence of blastopore (in contrast with the absence of blastopore in exogastrulae) provides the grounds on which “exogastrulation” is rejected. Alternatively, one might suggest, in emphasizing the *position* of the blastopore and treating *it* as the cue, the second researcher draws attention to the contrast between partial exogastrulae, in which the blastopore is in the “normal” position on the boundary of the embryo adjacent to the cite of partial involution, and the novel defect, on which the blastopore is located out on the protrusion. Either way, the important point is that the pattern of observation and reasoning that led researchers to reject “exogastrulation” would be analyzed as if it ranged over merely outwardly observable “cues” or “characteristics”—“x lacks characteristic y or perhaps has some feature that other non-x’s don’t have” (p. 12).

It is important not to conflate the pattern of observation and reasoning pursued by the researchers in our case study with that of the sort McConwell discusses. One can imagine a future in which xenogastrulation becomes a widely recognized defective phenotype and in which students are taught to identify xenogastrulation and distinguish it from exogastrulation in precisely the manner described above. Consulting an image of a xenogastrulae, an instructor might pose the question, “is this an exogastrula?” to a student. The student, without any other background knowledge, would be able to answer this question by simply recalling the “cue” they were taught in a lesson the day before: “No. Look at the position of the blastopore.” The pattern of observation and reasoning in such a case would lend itself well to analysis in McConwell’s terms, especially in light of the fact that the practice she describes is, in part, useful for students who presumably lack the kind of background knowledge that researchers in our case deployed in rejecting “exogastrulation.” But it is precisely the fact that this background knowledge was in fact operative in our case that makes such an analysis inappropriate.

To bring out how this background knowledge operated in our case study, consider again Griesemer’s (2018) description of the practice of marking and tracking: “scientists interact with a process by observing (“mental” marking by taking note) or experimenting (“physical” marking by perturbation or alteration) and thereby create “traces”: physical remnants that can be used as representations of the state of a marked process at multiple times.” From this point of view, in directing attention to the position of the blastopore, the researchers in our case mentally marked or “took note” of it. Insofar as this is the case, they treated the position of the blastopore as a “mark.” Then, they inferred from the marked position of the blastopore that, in these defective embryos, involution had occurred. This inference is an abductive one that developmental biologists can draw as readily as we can all infer fire from smoke. In other words, marking the position of the blastopore catalyzed the deployment of researchers’ background knowledge to identify “on the fly” the process *by means of which* the marker is produced, specifically involution. Once this explanation is in place, the blastopore is seen as a “physical remnant” that represents the state of the process of involution at a particular time *T*, where *T* = completion. In other words, at the conclusion of the inferential pattern catalyzed by marking it, the blastopore also serves as a *trace* that researchers use to *track* the process of involution.

This amounts to a recursive and distinctively explanatory inferential pattern that begins with researchers marking a feature, then proceeding to formulate an explanation for that marked feature in terms of some process (e.g. involution), and finally treating that very feature as a trace that represents the state of the process at a time. The inference is accomplished by deploying background knowledge to build “on the fly” an account of the process by means of which the mark/trace is produced. It was by means of this distinctively explanatory inferential pattern that, as we discussed above, researchers organized a conception of the phenomenon that assigned values to the temporal and spatial composition variables that Love (2018) discusses. It is precisely in this sense that we intend our main claim, namely, that the pattern of observation and reasoning that researchers in our case pursued in the immediate wake of their initial observations of the novel defect was one in which individuation and explanation were entangled.^15^

Returning to the key moment, after rejecting “exogastrulation,” one researcher proposed a name for the novel defect: “xenogastrulation.” Even though they had distinguished the novel defect from exogastrulation by way of the entangled pattern of observation and reasoning described above, the question of “what is xenogastrulation?” remained open insofar as researchers had yet to formulate a positive answer. Thus, one might be tempted to say that, for researchers at this moment in the case study, “xenogastrulation” meant nothing more than “not normal or exogastrulation.” Although intuitive, this way of unpacking the meaning of this term (at the time) would fail to take advantage of the analysis as we have developed it so far. Indeed, in light of our analysis, we can discern that, even as a “placeholder,” as we called it above, the term “xenogastrulation” was semantically much richer than such a merely negative explication of it would suggest.

As we saw above, the pattern of observation and reasoning that led researchers to reject “exogastrulation” involved making distinctively explanatory inferences regarding the means by which the marked, outwardly observable features of the defective embryos are produced. This was key to understanding the sense in which individuation and explanation were entangled in that pattern of observation and reasoning. Taking this into account, we can unpack the meaning of “xenogastrulation” in correspondingly entangled terms. We suggest that, as a placeholder, “xenogastrulation” inherited content from both the individuative and explanatory aspects of the pattern of observation and reasoning that led to its proposal. Specifically, we propose that “xenogastrulation” meant *some sort of pathological phenotype of such an outward character that it must be brought about by some means other than those by which exogastrulation is.*

Terms that function as “placeholders” in this way are not uncommon in science (Nathan, 2021).^16^ To give another example, in early research on cellular motility phenomena, researchers labeled the odd behavior of certain cellular particles “saltatory motion”—“sudden excursions of cytoplasmic particles over distances to extensive to be *accounted for* as Brownian motion” (Allen & Kamiya, 1964; emphasis added). As a term, “saltatory motion” was formulated long before it was understood that motor proteins like myosin, kinesin and dynein drive it by carrying particles along cytoskeletal filaments. Nonetheless, even lacking a positive account of the means by which these particles move in the characteristic way they do, the content of the term “saltatory motion” was not limited merely to the outward character of the motion. It encompassed the means by which the motion is produced—*particle motion of such an outward character that it must be brought about by means other than those by which Brownian motion is, specifically, the action of thermal forces.*

### What it* is*

The pattern of observation and reasoning just discussed was sufficient to individuate the defect as a genuinely novel form of pathological development deserving of its own term, “xenogastrulation*.*” As we just argued, the content of this term inherited aspects from both of the entangled individuative and explanatory aspects of the pattern of observation and reasoning that led to its proposal. “Xenogastrulation” constituted a tentative answer to both the individuative (what is it?) and explanatory (by what means does it occur?) questions: *a pathological phenotype of such an outward character that it must be brought about by some means other than those by which exogastrulation is.* We turn now to analyze the pattern of observation and reasoning that led to the formulation of a positive, empirically tractable answer to the question “what is xenogastrulation?” that, at the same time, provided a tentative answer to the question of “by what means does xenogastrulation come about?”.

The first proposed answer—“convergent extension of the whole embryo”—was inspired by the fact that the experimental interventions that produced xenogastrulation were conducted on proteins known to affect convergent extension. Additionally, the embryos exhibiting the defect were longer and thinner than typical gastrulae, suggesting that perhaps they come to have their characteristically distorted morphology by means identical to those by which notochords themselves come to be long and thin (i.e., convergent extension). As we noted above, researchers rejected this proposal in favor of “a mechanical distortion of the whole embryo due to hyperactive convergent extension of dorsal mesoderm.”

While researchers understood both as answers to the individuative question, “what is xenogastrulation?”, what made the latter answer preferable is that, unlike the former, it proposes clear *explanatory* relationships between the elements of the defective embryos. The latter description specifies that the dumb-bell shape of a xenogastrula might be explained as a *mechanical* distortion *due to* particular behaviors of a particular tissue. This suggested a path forward for further empirical investigation: consulting old ISH data to see if marked notochord indeed shows evidence for hyperactive convergent extension in xenogastrulae. The first proposal, “convergent extension of the whole embryo,” is much less clear on this explanatory score. After all, convergent extension is a process that occurs over the localized cell population that constitutes dorsal mesoderm rather than a process that occurs across an entire embryo. Does “convergent extension of the whole embryo” imply merely that the embryo’s shape constricts and elongates as dorsal mesoderm does, or does it imply that the cell-migratory behaviors by which convergent extension occurs in dorsal mesoderm have somehow become de-localized and distributed throughout the whole embryo? And, if the latter, what *precisely* should one expect to see when closely observing the behaviors of cells outside the dorsal mesoderm? “A mechanical distortion of the whole embryo due to hyperactive convergent extension,” on the other hand, spatially localizes the process of convergent extension to the dorsal mesoderm, and it appeals to that process as an explanatory factor in terms of which to give an account of the means by which the embryo as a whole comes to have its characteristically distorted morphology.

In philosophical terms, “convergent extension of the whole embryo” is ambiguous in how it assigns values to the spatial composition variable that Love (2018) discusses. Convergent extension is a process localized to a *tissue*—dorsal mesoderm—but the formulation, confusingly, de-localizes it from that spatial scale and distributes it across the spatial scale characteristic of the *embryo as a whole*. “A mechanical distortion of the whole embryo due to hyperactive convergent extension” resolves this spatial ambiguity by re-localizing convergent extension to mesoderm, “*where* it belongs.”

However, we emphasize that it was not concern with spatial relationships per se that motivated the researchers’ reasoning in this instance. Reflecting our general claim that individuation and explanation were entangled in this pattern of reasoning, the conceptual clarification of spatial relationships was important because it allowed “convergent extension” and “the whole embryo” to be brought into a sensible *explanatory* relation. After all, what the researchers sought was a positive answer to the question of “what is xenogastrulation?” where “xenogastrulation” means, specifically, *some sort of pathological phenotype of such an outward character that it must be brought about by some means other than those by which exogastrulation is.* They settled on “a mechanical distortion of the whole embryo due to hyperactive convergent extension” as a viable hypothesis for the reason that it provided a positive, albeit tentative, account of those means. Future candidate answers to the question, “what is xenogastrulation?” will, no doubt, also have to obey this explanatory constraint.

## Conclusion

We have offered a philosophical analysis of the pattern of observation and reasoning by which researchers individuated a novel developmental defect, both by distinguishing it from what it is *not* and by formulating a positive conception of it to facilitate further empirical research.

In developing our analysis, we identified a distinctive form of the practice of marking and tracking. In the case of these researchers, marking a feature of the phenomenon catalyzed the deployment of practitioners’ background knowledge in drawing abductive inferences to the process by means of which the marked feature is produced. In this manner, the marked feature also served as a trace, i.e., a “physical remnant” that represents the very process that the abductive inference identifies as the process that produces the mark/trace. By these explanatory means, researchers formulated a spatiotemporally rich conception of the novel defect that assigned values to a constellation of spatial and temporal variables. Thus, the pattern of observation and reasoning that their marking and tracing practice supported, and by which they distinguished the novel defect from exogastrulation, is one in which individuation and explanation are entangled.

This entanglement of individuation and explanation persisted after researchers dubbed the novel defect “xenogastrulation” and attempted to formulate a positive answer to the question of “what is xenogastrulation?” As a “placeholder,” the term “xenogastrulation” inherited content from both the individuative and explanatory dimensions the pattern of observation and reasoning that led to its proposal—*some sort of pathological phenotype of such an outward character that it must be brought about by some means other than those by which exogastrulation is.*

It is unsurprising, then, that researchers sought to answer “what is xenogastrulation?” in terms that would specify a positive proposal as to the means by which it comes about. The fact that it was induced as a consequence of intervening on molecules known to be causally implicated in convergent extension, plus the fact that the defective embryos exhibited a characteristically long and thin morphology, led researchers to formulate “convergent extension of the whole embryo.” But recognizing that this did not place “convergent extension” and “the whole embryo” in an intelligible *explanatory* relation to one another prompted the search for an alternative that would. We reiterate that it was specifically the concern to put them in an appropriate *explanatory,* as opposed to *spatial*, relationship to one another that drove the shift from the first formulation to the second. It was the drive to explain rather than a drive to spatially organize that motivated the shift. An appropriately spatially organized conception of the novel defect was a consequence of this shift, but not the reason for its occurrence. This is evidenced by the fact that what they sought was a positive answer to the question of “what is xenogastrulation?” where “xenogastrulation” means, specifically, *some sort of pathological phenotype of such an outward character that it must be brought about by some means other than those by which exogastrulation is.* They settled on “a mechanical distortion of the whole embryo due to hyperactive convergent extension” for the reason that it provided positive, albeit tentative, account of those means that they knew how to vet empirically by going back to their old ISH data.^17^

The old ISH data they consulted to vet this formulation did not speak in favor of it. To this extent, the question “what is xenogastrulation?” remains open and, in light of the fact that the pattern of observation and reasoning characteristic of their attempts to answer this question was one in which questions of individuation and explanation were entangled, it will remain so until further empirical research into the means by which xenogastrulation occurs provides researchers with a more robust explanatory account.

So, were researchers in this case trying to distinguish the novel phenotype from other objects (e.g. exogastrulation) and formulate a positive conception of it that would facilitate identification of xenogastrulae? That is, was the pattern of observation and reasoning they pursued over the course of the case study aimed at *individuating* the phenomenon? Or, in these key moments, were they speculating about the means by which the novel defect came about and attempting to formulate positive, albeit tentative, accounts of those means? In short, were they individuating it, or explaining it?

The “key moment” involving noting the position of the blastopore indicates that researchers were ambiguously doing both in the same breath. In the understanding of both developmental biologist interlocutors, the remark, “note the position of the blastopore” meant “look, this isn’t exogastrulation, because it comes about *by means* other than a failure of involution.” The researcher who initially marked the position of the blastopore indeed used the marked feature as device for individuating the novel defect from exogastrulation but, at the same time, used it as a device for inviting interlocutors to recognize that the means by which this novel phenotype came about, whatever they may be exactly, are not those by which exogastrulation does. The pattern of observation and reasoning here is positively ambiguous with respect to whether it was individuative or explanatory. It was inseparably both at the same time. The contrast between this pattern of observation and reasoning and that involved the pattern McConwell (2024) analyzes in her discussion of “cues” illustrates how the pattern in our case involved a distinctively explanatory dimension and throws into relief the ambiguity characteristic of the pattern we analyze.

Once the researchers determined that the phenomenon was not exogastrulation because it does not come about by the same means, they asked, if not exogastrulation, then what it is? Their initial answer was “convergent extension of the whole embryo.” But, they noted, this did not place the process of convergent extension in an *explanatory* relation vis-à-vis the whole embryo that would facilitate vetting it as a proposal. In other words, they rejected it as an answer to the question “what is it?” (Or, is the question “by what means does it occur? *Both*, and that is precisely our point) and landed on “a mechanical distortion of the whole embryo due to hyperactive convergent extension.” The ambiguity between individuation and explanation is patent in this formulation. What is it? A distortion of the whole embryo. By what means does it come about? Perhaps by means of distinctively mechanical (as opposed to, say, electrical) forces produced by hyperactive convergent extension in notochord. Answers to both questions are blended together in this single formulation that inherited its entangled content from the entangled pattern of observation and reasoning that generated it.

Finally, as we noted in our introduction, the practice of individuation is intimately related to broader scientific practices of description and characterization. A full telling of the implications of our analysis for the literature we discussed above on characterization and explanation is beyond what we can discuss here. We do wish to make two points in this regard, however.

First, we noted above that while philosophical views regarding the relationship between characterization and explanation vary, a common assumption is that the two are analytically distinguishable forms of scientific work. Again, we do not mean to challenge this general claim. However, insofar as our thesis that the work of individuation and explanation were entangled in the pattern of observation and reasoning that our researchers pursued can be transposed into the corresponding thesis that the work of *characterization* and explanation were so entangled in our case study, our case represents an exception to a basic philosophical assumption animating analyses of scientific practice.

Second, and more speculatively, it might constitute a quite significant exception. It might generally be the case that the scientific work that immediately follows in the wake of the initial observations of a novel phenomenon is of such an entangled, ambiguous form, either with respect to characterization and explanation or individuation and explanation (or both). Over the course of this entangled work, the ambiguity might get resolved, bifurcating the practice into a form more readily amenable to an analysis on which individuation (or characterization) work *is* distinguishable from explanation work. In our case study, for example, the formulation “a mechanical distortion of the whole embryo due to hyperactive convergent extension” suggests that some of the ambiguity was, by this point in the case, resolved such that we can distinguish between an *explanandum* (distorted embryo) and an *explanans* (hyperactive convergent extension) at which characterization and explanation work can be aimed, respectively. In other words, our analysis might be illustrative of how the kind of scientific work that has been analyzed in the literature on characterization and explanation—work that is amenable to analysis in terms of a clear distinction between the two—might originate out of an initially ambiguous phase of scientific inquiry and eventually come to be pursued separately, perhaps even by people in different labs or, as Dresow and Love (2025) emphasize, people in different disciplines. From this point of view, our case study can be understood as illustrating the early origins of scientific inquiry—a nascent phase at which efforts to individuate, characterize or explain have yet to be disentangled into distinguishable lines of scientific activity. We leave developing this suggestion for further work.

## Data Availability

Not applicable.

## Footnotes

1 A reviewer suggests that what we develop here is a “... possibly novel account of what it means to develop a ‘prepared description.’” According to Cartwright (1983), a prepared description “present[s] the phenomenon in a way that will bring it into the theory.” We do not follow up on that suggestion here for reasons of space but note it as in interesting suggestion for future work.

2 Freund & Grandy (2025) distinguish between *metaphysical* sortalism and *psychosortalism.* The metaphysical version seeks to identify “metaphysical conditions for the identity, individuation, and essence of entities” while psychosortalism is the view that human perceptual individuation of entities requires possession of a sortal concept that specifies the criteria for identifying an entity,” Psychosortalism is the view we mean to discuss only briefly here for the purpose of providing what we think is a useful foil against which the pattern of observation and reasoning we seek to analyze might be thrown into helpful relief.

3 Neither is it the case that the pattern of observation and reasoning these researchers pursued is one that could be attributed to animals like cats and dogs. In fact, as we will see, it is a pattern that could be plausibly attributed only to select humans that have the relevant background knowledge in developmental biology to support it.

4 This formulation on which explaining a phenomenon is a matter of specifying the means by which it occurs is partially inspired by Bollhagen (2021b) which argues that some understanding of the means by which the characteristic blackness of ravens comes to be is involved in our understanding of sentences like “ravens are black.” Thus, even if, in this sentence, “blackness” is understood as a feature that plays a role in individuating ravens from other birds, this is entangled, in the sense we intend in this paper, with what Bollhagen would call the “means-involving” content of the sentence.

5 The pattern of observation and reasoning pursued by the researchers in our case produced a tentative answer to the *explanatory* question, “*by what means* does xenogastrulation occur?” However, it also produced an answer to “*what is* xenogastrulation?” that specifies a *type* of which the particular embryos they observed would represent *tokens.* Inasmuch as this is the case, we are concerned with “type” individuation—how researchers individuated their novel defective embryos as tokens of a yet-to-be-formulated type, one that they worked (incompletely) to specify. Discussing individuation at the level of “type,” as opposed to particular individuals is not unprecedented. Havstad (2015), for instance, discusses “the classificatory practice of protein individuation: the sorting of individual token proteins in protein types, or kinds” (p. 75). In this way, our analysis intersects with broader philosophical issues regarding classification in biology. As McConwell (2024) notes, “individuation occurs in biology, but also concerns classification more generally.” We agree with her that “[t]here ought to be more cross contact between philosophy of biology literature on individuation and literature on classification...” and we suggest that our case study could potentially contribute to establishing such cross contact. We leave this for future work.

6 We thank an anonymous reviewer for encouraging us to relate our analysis here with philosophical discussions of characterization and explanation.

7 We note that Dresow & Love (2025) do not think that there is actually a “received view” in the philosophical literature on the nature of characterization but follow them in labeling the view that it is done for the sake of explanation “the received view.”

8 In spite of these differences between our use of the term and Dresow & Love’s, we preserve the term “entanglement” rather than introducing a new term because we think it is a useful way to situate the phenomenon we are analyzing alongside adjacent phenomena of “entanglement” discussed in recent philosophical scholarship. In addition to Dresow and Love (2025), we have in mind Cartwright et al., (2023) and Griesemer & Gerson (submitted), who discuss the “entanglement” of administrative and epistemic activities in the conduct of research in much the same spirit as we intend. Specifically, they show through careful, moment-to-moment analysis of a meeting of interdisciplinary researchers how, for key moments in their exchange, it is ambiguous as to whether the goal of the exchange was to set the administrative agenda for their next meeting or make epistemic progress toward a conceptual goal.

9 As we discuss, the novel defective phenotype that the researchers of the Merzdorf lab observed also involves a distortion of the embryo into a dumb-bell shape, which was the major motivation for the initial, albeit swiftly rejected, suggestion that the novel phenotype represents “a strange form of exogastrulation.”

10 For more on the role of conceptions of normal development in experimental embryology, see Lowe (2016).

11 We will save a detailed description of methods for future scientific reports. Here, we will only note that the embryos in Fig. 5B were injected with morpholino oligonucleotides to knock down the expression of two proteins: Aqp3b, an aquaglyceroporin that facilitates transport of water and glycerol through cell membranes, and PLD1, a phospholipase. These embryos were also injected with exogenous mRNAs to increase the expression of PKC, a protein kinase. All three of these proteins are known to affect convergent extension through interactions with non-canonical Wnt pathways (Forecki et al., 2018; Lee et al., 2016; Wallingford et al., 2001).

12 The name was chosen both as a recognition of the strangeness of the phenomenon (the prefix “xeno” derives from the Ancient Greek for “strange”) and as an homage to the research organism, *Xenopus laevis*. This usage therefore does not invoke the sense of “transplanted from a different species or different organism,” with which the prefix “xeno” is often associated, although both uses share a common etymology.

13 Using their old ISH data in the new context of evaluating the proposal that xenogastrulation is “a mechanical distortion of the whole embryo due to hyperactive convergent extension” is an example of what Griesemer and Barragán (2022) describe as *re-situation.* Thanks to Jim Griesemer for this point.

14 The embryo shown in Fig. 9A was injected with morpholino oligonucleotides to knock down the expression of Aqp3b. It was also injected with exogenous mRNAs to increase the expression of Aqp1, a traditional aquaporin that facilitates the transport of water (but not glycerol, unlike Aqp3b) through cell membranes.

15 While we do not present it here for purposes of space, the same analysis can be applied to the researchers *marking* the presence of neural plate which, like the position of the blastopore, served also as a *trace* by which they tracked the process of involution.

16 According to Nathan (2021), placeholders can function in two ways. As “frames,” they “spell out an explanandum; a behavior, or range of behaviors, in need to explanation. On the other hand, a placeholder may stand in for the mechanisms, broadly construed, which bring about the patterns of behavior specified by the frame regardless of how well their nature and structure are understood” (p. 18). Therefore, Nathan’s “placeholders” have, in our sense of the term, “entangled” individuative and explanatory content. “Xenogastrulation” is an example of a placeholder in this sense. In fact, a promising line of future research might look at the kinds of cases Nathan discusses to see if a pattern of observation and reasoning explicable along the lines we suggest here is involved in the formation of the placeholders he discusses.

17 As we mentioned in our introduction, our analysis bears on the literature on the relationship between characterization and explanation insofar as philosophical analyses of the former overlap with analyses of individuation practices. A full answer to the question of how our article bears on the characterization literature would necessitate an analysis of this overlap, something we do not have space for here but save for future work. We caution against a strong reading of the overlap which would justify a reading of the present article that substitutes “characterization” for each instance of the term “individuation.” Regarding the paragraph above, however, we will make one point, namely, that the fact that a more appropriate spatial *characterization* of the phenomenon resulted from the *explanatory* concerns discussed in this paragraph speaks in favor of our entanglement thesis as it would be formulated in terms of the relationship between characterization and explanation.
